# WIP1 promotes cancer stem cell properties by inhibiting p38 MAPK in NSCLC

**DOI:** 10.1038/s41392-020-0126-x

**Published:** 2020-04-15

**Authors:** Kaiyuan Deng, Liang Liu, Xiaoming Tan, Zhen Zhang, Jianjun Li, Yang Ou, Xin Wang, Shuang Yang, Rong Xiang, Peiqing Sun

**Affiliations:** 1 School of Medicine, Nankai University, Tianjin, 30071 China; 2 Departments of Immunology and Microbial Science, The Scripps Research Institute, La Jolla, CA 92037 USA; 3 Department of Cancer Biology and Wake Forest Baptist Comprehensive Cancer Center, Wake Forest School of Medicine, Winston-Salem, NC 27157 USA; 4 Department of Respiratory Disease, South Campus, Renji Hospital, School of Medicine Shanghai Jiaotong University, Shanghai, China

**Keywords:** Cancer stem cells, Lung cancer, Cancer stem cells, Lung cancer

## Abstract

Cancer stem cells (CSCs) are a small population of stem cell-like cancer cells that can initiate tumors in vivo, and are the major source of cancer initiation, relapse, and drug resistance. We previously reported that the p38 MAPK, through its downstream effectors MK2 and HSP27, suppressed CSC properties by downregulating the expression of transcription factors that mediate stemness in non-small-cell lung cancer (NSCLC) cells, and that despite unaltered total expression of total p38 proteins, the levels of activated p38 were reduced in NSCLC tissues. However, the mechanism underlying the reduced levels of activated p38 in NSCLC is unknown. In this study, we identified WIP1, a p38 phosphatase frequently overexpressed in cancer, as a suppressor of p38 in a pathway that regulates CSC properties in NSCLC. Increased WIP1 expression correlated with reduced levels of activated p38, and with increased levels of a CSC marker in NSCLC tissues. Further investigation revealed that WIP1 promoted stemness-related protein expression and CSC properties by inhibiting p38 activity in NSCLC cells. WIP1 inhibitors are currently under development as anticancer drugs based on their ability to reactivate p53. We found that a WIP1 inhibitor suppressed stemness-related protein expression and CSC properties by activating p38 in NSCLC cells in vitro and in vivo. These studies have identified the WIP1–p38–MK2–HSP27 cascade as a novel signaling pathway that, when altered, promotes CSC properties in NSCLC development, and have defined novel mechanisms underlying the oncogenic activity of WIP1 and the anticancer efficacy of WIP1 inhibitors.

## Introduction

Lung cancer is a leading cause of cancer-related death worldwide.^1^ It is divided into small-cell lung carcinoma and non-small- cell lung carcinoma (NSCLC).^2^ Since over 85% of lung cancer cases are NSCLC, which is associated with poor survival,^3^ it is of great importance to identify the underlying mechanisms, and to develop novel and effective therapies for NSCLC.

Cancer stem cells (CSCs), also termed tumor-initiating cells (TICs), are a small population of stem cell-like cancer cells that can initiate tumors in vivo.^4^ CSCs are the major source of cancer initiation, relapse, and drug resistance.^4^ The stemness-related proteins SOX2, OCT4, NANOG, KLF4, and c-Myc induce pluripotency in somatic cells,^5^ and also contribute to tumorigenesis^5–16^ and CSCs^6–10^ in cancers, including NSCLC. SOX2 has increased expression and promotes tumorigenesis in lung, breast, ovarian, and prostate cancers,^6,7,9–14,17–19^ and is required for CSCs and tumorigenesis in NSCLC.^8^

The p38 MAPK pathway was initially identified as a mediator of inflammation and stress responses, and was later found to regulate other conditions, including cancer.^20,21^ The role of p38 in cancer is context dependent. While some reported that p38 promotes tumorigenesis by mediating cancer cell invasion and metastasis,^22^ we and others have shown that the p38 pathway suppresses cancer development by inhibiting cell proliferation and mediating cellular senescence.^21,23^ p38 is a tumor suppressor in the lungs, as p38 deletion in adult mice promotes K-Ras^G12V^-induced lung cancer,^24^ but the mechanism underlying this observation and those underlying the tumor-suppressing activity of p38 in general are not completely understood. We recently reported that p38, especially the γ and δ isoforms of p38, suppresses the expression and reduces the stability of the stemness-related proteins SOX2, OCT4, NANOG, KLF4, and c-Myc and CSC properties via MK2-mediated HSP27 phosphorylation in NSCLC cells.^25^ Supporting a role of p38 in suppressing NSCLC development by inhibiting CSCs in vivo, we previously observed reduced levels of phosphorylated/activated p38 (p-p38), along with increased expression of SOX2, in NSCLC tissue samples compared with normal lung tissue samples. However, the mechanism underlying the reduced activation of p38 in NSCLC is unknown.^25^

WIP1 (PPM1D) is a type 2C Ser/Thr phosphatase (PP2Cδ) with oncogenic activities that exhibits amplification and overexpression in human cancers.^26^ WIP1 has multiple substrates, including p38, p53, MDM2, and DNA damage response mediators (ATM, Chk1, and Chk2). The oncogenic activity of WIP1 has been attributed to suppression of p53 through dephosphorylation of p53-Ser15, a site critical for p53 activation, MDM2, which promotes the degradation of the p53 protein, or ATM, Chk1, and Chk2, which are upstream kinase activators of p53. WIP1 inhibitors, including GSK2830371, are now under development as anticancer drugs based on their ability to inhibit tumor growth and sensitize cancer cells to chemotherapy by reactivating p53.^27–29^ However, neither WIP1 nor the therapeutic effect of WIP1 inhibitors has previously been linked to the properties of CSCs, which are the major source of cancer initiation, progression, relapse, and drug resistance. In this study, we identified WIP1 overexpression as a potential mechanism responsible for reduced p38 activity and enhanced CSC properties in NSCLC. Further studies demonstrated that WIP1 indeed induced stemless-related proteins and CSC properties by inhibiting p38 in NSCLC cells in vitro and in vivo, and that the WIP1 inhibitor GSK283071 activated p38 and suppressed stemness-related protein expression and CSC properties in a p38-dependent manner in NSCLC cells. These studies revealed novel genetic alterations, along with the associated signaling pathway, that promote CSC properties in NSCLC development, and identified novel mechanisms underlying the oncogenic activity of WIP1 and the anticancer efficacy of WIP1 inhibitors, which may expand the therapeutic application of WIP1 inhibitors.

## Results

### The expression of WIP1 is upregulated and negatively correlated with reduced levels of activated p38 in NSCLC

**Fig. 1:**
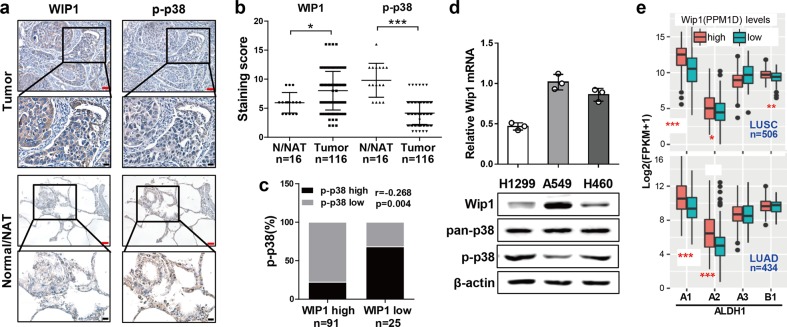
WIP1 expression is increased in NSCLC tissues, and this increased expression correlates with reduced levels of activated p38 and increased levels of the CSC marker ALDH1. **a** Representative images of IHC staining for WIP1 and phospho-p38 in serial sections of a human NSCLC tissue array containing 116 tumor tissue samples and 16 normal/normal adjacent (N/NAT) lung tissue samples. Red scale bar, 50 μm. Black scale bar, 20 μm. **b** Quantification of the IHC staining scores for WIP1 and p-p38 in the tissue array described in (**a**). The lines represent the quartiles and mean values of the tumor group (*N* = 116) or N/NAT group (*N* = 16). * indicates *P* < 0.05, and *** indicates *P* < 0.001 vs. the N/NAT control determined by Student’s *t* test. **c** Spearman rank correlation analysis indicating a negative correlation between WIP1 and p-p38 levels based on the IHC staining results in 116 tumor tissues (*r* = − 0.268, *p* = 0.004). **d** Relative mRNA (top panel) and protein (bottom panels) levels of WIP1, phospho-p38, and p38 in three NSCLC cell lines (H1299, A549, and H460) detected by qRT-PCR and Western blotting, respectively. The data are presented as the mean ± SD of three independent experiments. **e** TCGA data for the two major subtypes of NSCLC, lung squamous cell carcinoma (LUSC, top panel, *N* = 506) and lung adenocarcinoma (LUAD, bottom panel, *N* = 434) obtained from the cbioportal (www.cbioportal.org). The patients in each dataset were divided into WIP1-high (the top 25%, ≥75%) and WIP1-low (the bottom 25%, ≤25%) subgroups. The Mann–Whitney test was applied to assess the correlations between the expression levels of WIP1 and ALDH1 isoforms (A1–A3 and B1). * indicates *P* < 0.05, ** indicates *P* < 0.01, and *** indicates *P* < 0.001 determined by the Mann–Whitney test. The number of patients is indicated

Inactivation of the p38 pathway promotes cancer development in mice.^30–32^ Consistent with the tumor-inhibiting effect of p38 on human cancer, we previously reported that the level of activated/phosphorylated p38 (p-p38) was reduced in NSCLC tissue samples compared with normal/normal adjacent lung tissue samples (N/NAT).^25^ To explore the mechanism responsible for the downregulation of p38 activity in NSCLC, we investigated the relationship between WIP1, a p38 phosphatase overexpressed in cancer, and p-p38. We first performed immunohistochemistry to examine the expression of WIP1 in human lung cancer samples with a tissue array containing 116 NSCLC tumor tissue samples (60 squamous cell carcinoma and 56 adenocarcinoma specimens) and 16 normal lung tissues/normal adjacent lung tissue samples (N/NAT) (4N and 12 NAT specimens) (Fig. 1a). We found that WIP1 expression was significantly increased in the lung cancer tissue samples compared with the noncancerous tissue samples, which is consistent with the tumor-promoting role of WIP1 in human cancers (Fig. 1b). We also analyzed the expression of phosphorylated/activated p38 (p-p38), and investigated the relationship between WIP1 and p-p38 in NSCLC using the same tissue array (Fig. 1a). The results showed that the level of p-p38 was significantly lower in the tumor tissue specimens than in the normal tissue specimens (Fig. 1b), as we reported previously.^25^ Next, we divided the 116 NSCLC tumor tissue samples into two groups (high and low) based on their immunohistochemical staining scores for WIP1. The percentages of cases with high or low p-p38 levels were calculated by their staining scores in each group. By performing Spearman’s rank correlation test, we observed a significant negative correlation between WIP1 and p-p38 expression levels (*r* = − 0.268, *p* = 0.004) in lung cancer tissue (Fig. 1c).

The negative correlation between the expression level of WIP1 and the activation of p38 was also observed in NSCLC cell lines. We compared the expression of WIP1 and p-p38 in three NSCLC cell lines (H1299, A549, and H460) by Western blotting. Among these three cell lines, we observed the highest level of WIP1 and the lowest level of p-p38 in A549 cells, while H1299 cells had the lowest level of WIP1 but the highest level of p-p38. Moreover, H460 cells had intermediate levels of both WIP1 and p-p38 (Fig. 1d). These findings suggest that upregulation of WIP1 expression is at least partly responsible for the reduced phosphorylation and inactivation of p38 in NSCLC tissues and cell lines.

### Increased expression of WIP1 correlates with increased levels of the CSC marker ALDH1 in NSCLC

We previously demonstrated that reduced p-p38 levels correlated with increased SOX2 levels in NSCLC tissues, and that activated p38 suppressed CSC properties in NSCLC cell lines.^25^ To investigate whether the WIP1-mediated suppression of p38 activity contributes to CSC properties, we determined the relationship between WIP1 expression and levels of ALDH1, which is a well-established CSC marker in NSCLC,^33^ by analyzing data from The Cancer Genome Atlas (TCGA) database. We divided the cases for the two major NSCLC subtypes, lung adenocarcinoma (LUAD) and lung squamous cell carcinoma (LUSC), in the TCGA into WIP1-high (the top 25%) and WIP1-low (the bottom 25%) groups, and compared the expression levels of the four ALDH1 isoforms (A1–A3 and B1) between the two groups. The analysis revealed associations between high WIP1 expression and increased levels of ALDH1A1 and ALDH1-A2 (Fig. 1e) in both LUAD and LUSC. These results suggest that suppression of the phosphorylation and activation of p38 by increased WIP1 levels may enhance CSC properties in NSCLC.

### WIP1 promotes the expression of stemness-related proteins and CSC properties in NSCLC cells

**Fig. 2:**
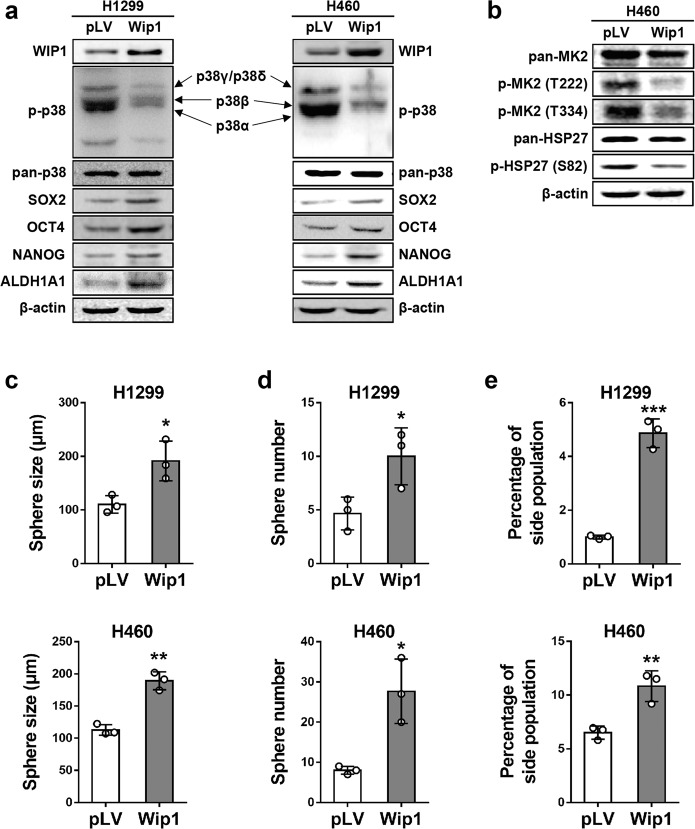
Ectopic expression of WIP1 reduces the levels of activated p38 and enhances stemness-related protein expression and CSC properties in NSCLC cells. **a** Western blotting was used to analyze the expression of WIP1, phospho-p38, p38, SOX2, OCT4, NANOG, and ALDH1A1 in H1299 (left panels) and H460 (right panels) cells transduced with a WIP1-overexpressing plasmid (WIP1) or vector control (pLV). Arrows indicate the positions of p38 isoforms. **b** Western blotting was used to analyze MK2, phospho-MK2 (Thr222), phospho-MK2 (Thr334), HSP27, and phospho-HSP27 (Ser82) in H460 cells transduced with the WIP1-overexpressing plasmid (WIP1) or vector control (pLV). **c**, **d** A sphere formation assay was performed with H1299 (top graphs) and H460 (bottom graphs) cells transduced with the WIP1-overexpressing (WIP1) or vector control (pLV) plasmid. Quantifications of sphere sizes (**d**) and numbers (**e**) are shown in bar graphs. The data are presented as the mean ± SD of three independent experiments. * indicates *P* < 0.05, and ** indicates *P* < 0.01 vs. the pLV control determined by Student’s *t* test. **e** The percentage of the side population was measured by flow cytometry following Hoechst 33342 staining of H1299 (top graph) and H460 (bottom graph) cells transduced with the WIP1-overexpressing (WIP1) or vector control (pLV) plasmid. The bar graphs show the quantifications of the percentages of the side population. The data are presented as the mean ± SD of three independent experiments. ** indicates *P* < 0.01, and *** indicates *P* < 0.001 vs. the pLV control determined by Student’s *t* test

The correlation between increased WIP1 expression and increased expression of the CSC marker ALDH1 in NSCLC prompted us to investigate whether WIP1 regulates stemness-related protein expression and CSC properties in NSCLC cells. We used a lentiviral system to stably overexpress WIP1 in H1299 cells with low WIP1 levels (H1299-WIP1 cells), and H460 cells with intermediate WIP1 levels (H460-WIP1). Western blotting results demonstrated that overexpression of WIP1 significantly downregulated the level of phosphorylation of p38 isoforms, including p38α, p38β and p38γ, and/or p38δ, and increased the expression of stemness-related proteins, including SOX2, OCT4, and NANOG, and the expression of the CSC marker ALDH1A1 in both H1299 and H460 cells (Fig. 2a, Supplementary Data Fig. S1a). We could not differentiate the signal for phosphorylated p38γ from that for p38δ, as they migrated to the same position on the Western blots (Supplementary Data Fig. S1a). In addition, the overexpression of WIP1 in H460 cells effectively reduced the phosphorylation of MAPKAPK-2 (MK2) at Thr222 and Thr334, and the phosphorylation of HSP27 at Ser82 (Fig. 2b), which was consistent with our previous report showing that p38 suppresses the expression of stemness-related proteins through MK2-dependent phosphorylation of HSP27.^25^

We next assessed the impact of WIP1 overexpression on CSC properties using sphere formation and side population assays. We found that increased expression of WIP1 induced both H1299 and H460 cells to form larger (Fig. 2c, Supplementary Data Fig. S1b) and more (Fig. 2d, Supplementary Data Fig. S1b) spheres than vector control (pLV) treatment. Similar results were obtained with a side population assay that measured the percentage of cells showing increased efflux of the DNA-binding dye Hoechst 33342 by flow cytometry, which identifies CSCs.^24,34–38^ Compared with vector control treatment, ectopic expression of WIP1 led to a higher side population percentage in H1299 and H460 cells (Fig. 2e, Supplementary Data Fig. S1c).

**Fig. 3:**
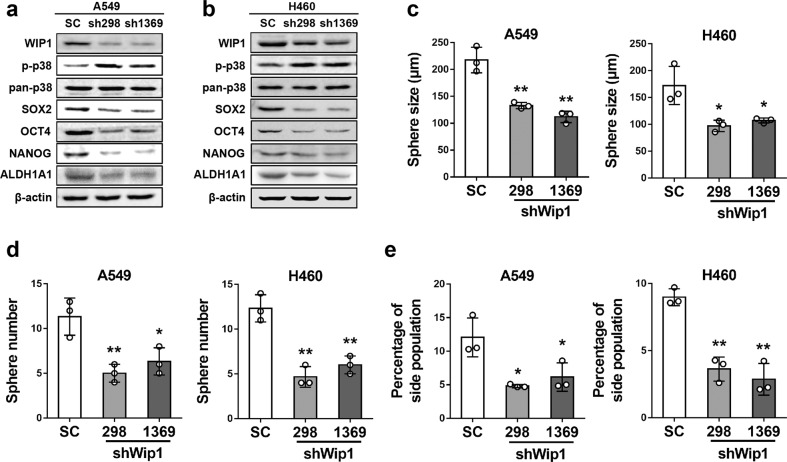
shRNA-mediated knockdown of WIP1 expression increases the levels of activated p38 and reduces stemness-related protein expression and CSC properties in NSCLC cells. **a**, **b** Western blotting of WIP1, phospho-p38, p38, stemness-related proteins, and ALDH1A1 in A549 (**a**) and H460 (**b**) cells transduced with WIP1 shRNAs (sh298 and sh1369) or a scrambled sequence control (SC). **c**, **d** Sphere formation assay performed with A549 (left graphs) and H460 (right graphs) cells transduced with WIP1 shRNAs (sh298 and sh1369) or a scrambled sequence control (SC). The bar graphs show the quantifications of sphere sizes (c) and numbers (d). The data are presented as the mean ± SD of three independent experiments. * indicates *P* < 0.05, and ** indicates *P* < 0.01 vs. the SC control determined by Student’s *t* test. **e** The percentage of the side population measured by flow cytometry following Hoechst 33342 staining of H1299 (left graph) and H460 (right graph) cells transduced with WIP1 shRNAs (sh298 and sh1369) or a scrambled sequence control (SC). The bar graphs show the quantifications of the percentages of the side population. The data are presented as the mean ± SD of three independent experiments. * indicates *P* < 0.05, and ** indicates *P* < 0.01 vs. the SC control determined by Student’s *t* test

In a reciprocal experiment, we stably knocked down WIP1 expression using two shRNAs in A549 cells with high WIP1 levels (A549-sh298 and A549-sh1369 cells) (Fig. 3a), and in H460 cells with intermediate WIP1 levels (H460-sh298 and H460-sh1369 cells) (Fig. 3b). Our results showed that knocking down WIP1 expression upregulated the levels of p38, reduced the levels of the stemness-related proteins SOX2, OCT4, and NANOG, and the CSC marker ALDH1A1, as determined by Western blot analysis (Fig. 3a, b), and decreased sphere formation (Fig. 3c, d, Supplementary Data Fig. S2a) and the side population percentage (Fig. 3e, Supplementary Data Fig. S2b) in both A549 and H460 cells compared with the vector control cells (SC).

Collectively, these findings indicate that WIP1 is both necessary and sufficient for the inhibition of p38 phosphorylation and the upregulation and/or maintenance of stemness protein expression and CSC properties in NSCLC cells.

### WIP1 promotes the CSC properties of NSCLC cells through inactivation of p38

Based on the ability of WIP1 to dephosphorylate and inactivate p38, and the correlations among increased WIP1 expression, reduced p-p38 levels, and increased CSC marker ALDH1 expression in NSCLC, we investigated the possibility that WIP1 promotes stemness-related protein expression and CSC properties by inhibiting p38.

MKK3 and MKK6 are upstream-activating kinases of p38. We analyzed the effect of WIP1 overexpression in H460 cells in which p38 was constitutively activated by an active mutant of MKK3 (MKK3E), and the effect of WIP1 knockdown in H460 cells in which p38 was inhibited by a dominant-negative mutant of MKK6 (MKK6A).

**Fig. 4:**
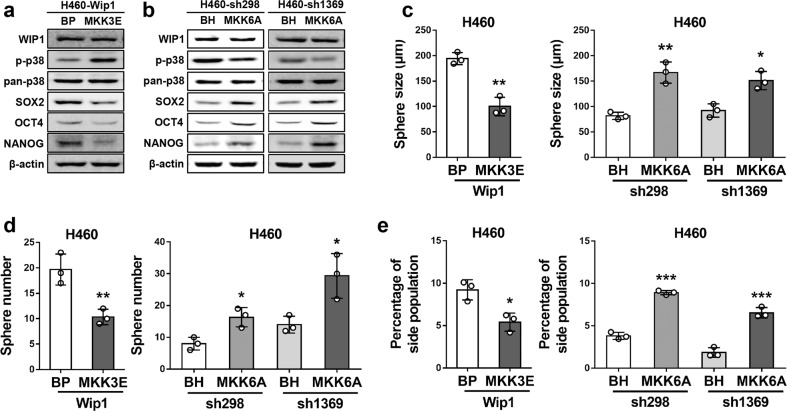
WIP1 promotes stemness-related protein expression and CSC properties by inhibiting p38 activation in NSCLC cells. **a** Western blotting of WIP1, phospho-p38, p38, and stemness-related proteins in H460 cells transduced with constitutively active MKK3 (MKK3E) or a vector control (BP) and WIP1. **b** Western blotting of WIP1, phospho-p38, p38, and stemness-related proteins in H460 cells transduced with dominant-negative MKK6 (MKK6A) or a vector control (BH) and shRNAs specific for WIP1 (sh298 and sh1369). **c**, **d** Bar graphs showing the quantifications of sizes (**a**) and numbers (**b**) of spheres formed by H460 cells transduced with MKK3E or the vector control (BP), and WIP1 (left graphs) and H460 cells transduced with MKK6A or the vector control (BH) and WIP1-specific shRNAs (sh298 and sh1369) (right graphs). The data are presented as the mean ± SD of three independent experiments. * indicates *P* < 0.05, and ** indicates *P* < 0.01 vs. the BP or BH control determined by Student’s *t* test. **e** The percentage of the side population measured by flow cytometry following Hoechst 33342 staining of H460 cells transduced with MKK3E or the vector control (BP), and WIP1 (left graph) and H460 cells transduced with MKK6A or the vector control (BH) and WIP1-specific shRNAs (sh298 and sh1369) (right graph). The data are presented as the mean ± SD of three independent experiments. * indicates *P* < 0.05, and *** indicates *P* < 0.001 vs. the BP or BH control determined by Student’s *t* test

Restoration of the WIP1-inhibited p-p38 level by MKK3E (Fig. 4a) led to decreases in the expression levels of the stemness-related proteins SOX2, OCT4, and NANOG, as determined by Western blot analysis (Fig. 4a), in the ability to form spheres (Fig. 4c, d, Supplementary Data Fig. S3a), and in the percentage of the side population (Fig. 4e, Supplementary Data Fig. S3b) compared with control H460 cells without MKK3E. In contrast, H460 cells with abrogation of the WIP1 knockdown-induced activation of p38 by MKK6A exhibited increases in SOX2, OCT4, and NANOG expression (Fig. 4b), the size and number of spheres formed by the cells (Fig. 4c, d, Supplementary Data Fig. S3a), and the percentage of the side population (Fig. 4e, Supplementary Data Fig. S3b) compared with control H460 cells without MKK6A-mediated inhibition of p38. The level of WIP1 was not changed by either MKK3E or MKK6A (Fig. 4a, b). These results indicate that p38 activation by MKK3E reverses the stimulatory effect of WIP1, and that p38 suppression by MKK6A reverses the inhibitory effect of WIP1 shRNAs on stemness-related protein expression and CSC properties, demonstrating that WIP1 acts upstream of p38 to promote stemness-related protein expression and CSC properties in NSCLC cells.

In cells with ectopic expression of both WIP1 and MKK3E, the p38 phosphorylation status depends on the balance of signaling strength between these two proteins with opposite effects on p38. In the presence of overexpressed MKK3E, it is highly likely that the overexpressed WIP1 does not completely dephosphorylate p38, leading to the rescue of the inhibition of CSC properties by MKK3E in Wip1-overexpressing cells.

### The WIP1 inhibitor GSK2830371 activates p38 and reduces stemness-related protein expression and CSC properties in NSCLC cells

The increased expression of WIP1 in NSCLC and its essential role in mediating CSC properties in NSCLC cells raise the possibility of treating NSCLC by targeting CSCs with WIP1 inhibitors. Indeed, WIP1 inhibitors, including GSK2830371 (GSK), are currently being pursued as anticancer drugs based on their ability to reactivate p53.^27–29^ We thus tested the effect of GSK on CSC properties in NSCLC cells.

**Fig. 5:**
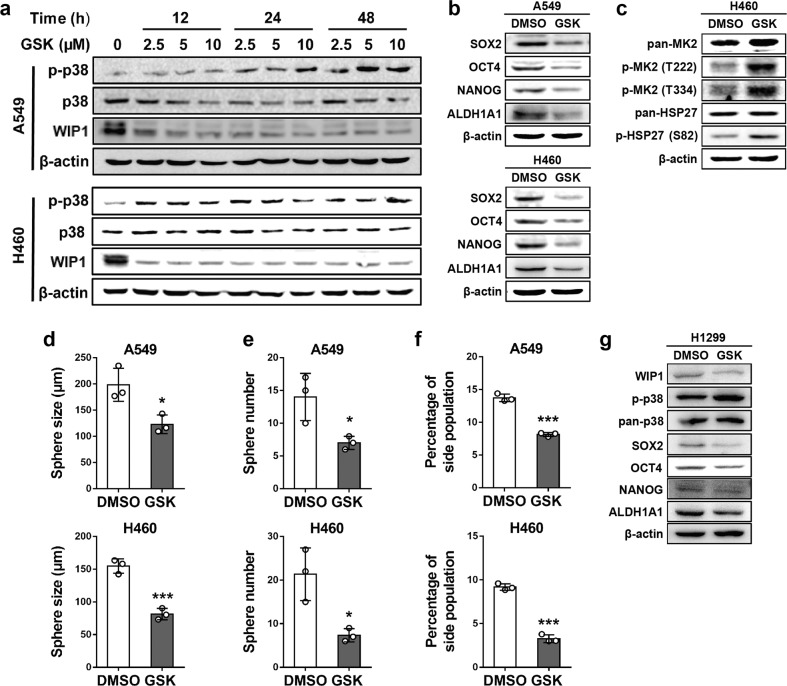
The WIP1 inhibitor GSK2830371 enhances p38 activation and suppresses stemness-related protein expression and CSC properties in NSCLC cells. **a** Expression of phospho-p38, p38, and WIP1 in A549 (top panels) and H460 (bottom panels) cells evaluated by Western blotting after treatment with the indicated concentrations of GSK2830371 or DMSO for 12, 24, or 48 h. **b** Western blotting of stemness-related proteins and ALDH1A1 in A549 (top panels) and H460 (bottom panels) cells treated with 10 μM GSK2830371 or DMSO for 48 h. **c** Western blotting of MK2, phospho-MK2 (Thr222), phospho-MK2 (Thr334), HSP27, and phospho-HSP27 (Ser82) in H460 cells treated with 10 μM GSK2830371 or DMSO for 48 h. **d**, **e** Quantifications of the sizes (**d**) and numbers (**e**) of spheres formed by A549 (top graphs) and H460 (bottom graphs) cells after incubating with 10 μM GSK2830371 or DMSO for 10 days. The data are presented as the mean ± SD of three independent experiments. * indicates *P* < 0.05, and *** indicates *P* < 0.001 vs. the vehicle control (DMSO) determined by Student’s *t* test. **f** The percentage of the side population measured by flow cytometry following Hoechst 33342 staining of A549 (top graph) and H460 cells (bottom graph) after a 48-h treatment with 10 μM GSK2830371 or DMSO. The data are presented as the mean ± SD of three independent experiments. *** indicates *P* < 0.001 vs. the vehicle control (DMSO) determined by Student’s *t* test. **g** Western blotting of stemness-related proteins and ALDH1A1 in H1299 cells treated with 10 μM GSK2830371 or DMSO for 48 h

In agreement with previous reports,^27–29^ GSK inhibited the activity and protein level of WIP1, and induced p38 activation (p-p38) in a time- and dose-dependent manner in A549 and H460 cells (Fig. 5a). Furthermore, GSK reduced SOX2, OCT4, and NANOG protein levels, the expression levels of the CSC marker ALDH1A1 (Fig. 5b), sphere formation (Fig. 5d, e, Supplementary Data Fig. S4a), and the percentage of the side population (Fig. 5f, Supplementary Data Fig. S4b) in both cell lines. We also found that treatment with GSK promoted the phosphorylation of the p38 downstream targets MK2 (Thr222 and Thr334) and HSP27 (Ser82) in H460 cells (Fig. 5c), indicating that GSK activates p38 signaling pathway components previously reported to suppress CSC properties.^25^ Moreover, in addition to producing these effects on A549 and H460 cells with the wild-type TP53 gene, GSK also reduced the expression levels of SOX2, OCT4, NANOG, and the CSC marker ALDH1A1 in H1299 cells with homozygous deletion of the TP53 gene (Fig. 5g). These results demonstrate that this WIP1 inhibitor, which activates p38, can reduce stemness-related protein expression and suppress CSC properties independent of p53 in NSCLC cells.

### WIP1 promotes and the WIP1 inhibitor GSK2830371 suppresses the tumor-initiating ability and CSC properties of NSCLC cells in a p38-dependent manner in mouse xenograft models

**Fig. 6:**
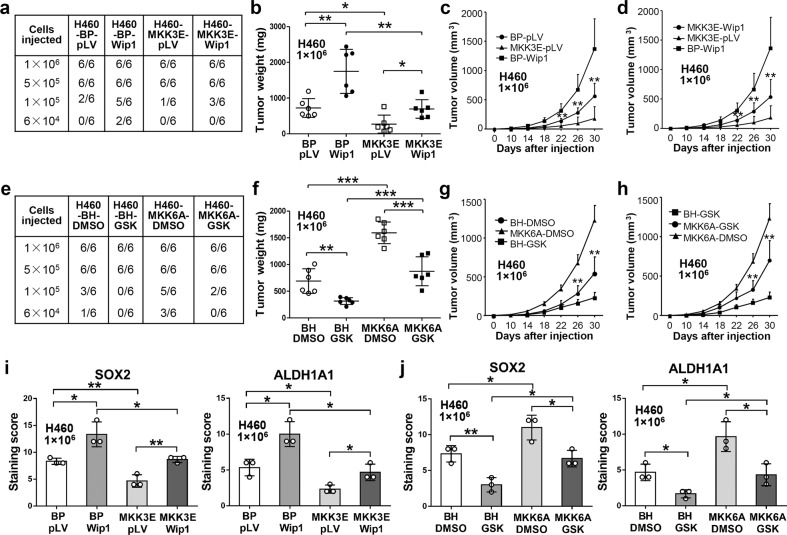
WIP1 promotes and the WIP1 inhibitor GSK2830371 reduces the tumor-initiating ability of NSCLC cells and the growth rate of xenograft tumors formed by NSCLC cells in p38-dependent manners. **a** Numbers of tumors formed after subcutaneous injection of six BALB/c nude mice with the indicated numbers of H460 cells transduced with MKK3E or a vector control (BP), and WIP1 or a vector control (pLV). **b** Tumor weights measured at the end of the study. The tumors were formed following injection of 1 × 10^6^ H460 cells transduced with MKK3E or the corresponding vector control (BP), and WIP1 or the corresponding vector control (pLV). The data are presented as the mean ± SD, and the lines represent the quartiles and mean values of six xenograft tumors from each group. * indicates *P* < 0.05, and ** indicates *P* < 0.01 vs. the control determined by Student’s *t* test. **c**, **d** The growth curves of xenograft tumors formed by injection of 1 × 10^6^ H460 cells transduced with MKK3E or the corresponding vector control (BP), and WIP1 or the corresponding vector control (pLV). The data are presented as the mean ± SD of six xenograft tumors from each group. ** indicates *P* < 0.01 vs. the control determined by Student’s *t* test. **e** Numbers of tumors formed after subcutaneous injection of six BALB/c nude mice with the indicated numbers of H460 cells transduced with MKK6A or a vector control (BH), and then treated with either DMSO or GSK2830371 (25 mg/kg via intraperitoneal injection once daily) from day 1 to 30. **f** Tumor weights at the end of the study. The tumors were formed by injection of 1 × 10^6^ H460 cells transduced with MKK6A or the vector control (BH), and treated with either DMSO or GSK GSK2830371 for 30 days. The data are presented as the mean ± SD, and the lines represent the quartiles and mean values of six xenograft tumors from each group. ** indicates *P* < 0.01, and *** indicates *P* < 0.001 vs. the control determined by Student’s *t* test. **g**, **h** The growth curves of xenograft tumors formed by injection of 1 × 10^6^ H460 cells transduced with MKK6A or the vector control (BH) after 30 days of treatment with either DMSO or GSK GSK2830371. The data are presented as the mean ± SD of six xenograft tumors from each group. ** indicates *P* < 0.01 vs. the control determined by Student’s *t* test. **i**, **j** Quantification of IHC staining scores for SOX2 and ALDH1A1 in the xenograft tumors described in (**b**) and (**f**), respectively. The data are presented as the mean ± SD of three xenograft tumors from each group. * indicates *P* < 0.05, and ** indicates *P* < 0.01 vs. a control determined by Student’s *t* test

The ability to initiate tumors is a key characteristic that defines CSCs. We thus measured the effect of WIP1 and the WIP1 inhibitor GSK2830371 on the tumor-initiating ability of H460 cells in mouse xenograft models using a limiting dilution assay performed by injecting varying amounts of cells into nude mice. We found that at multiple cell concentrations (e.g., 1 × 10^5^ and 6 × 10^4^), ectopic expression of WIP1 increased the frequency of tumor formation by H460 cells (Fig. 6a, Supplementary Data Fig. S5, Supplementary Table S1), while GSK reduced this frequency (Fig. 6e, Supplementary Data Fig. S6, Supplementary Table S1). Moreover, constitutive activation of p38 by MKK3E reversed the stimulatory effect of WIP1 on tumor initiation (Fig. 6a, Supplementary Data Fig. S5, Supplementary Table S1), and inactivation of p38 by MKK6A reversed the inhibitory effect of GSK on tumor initiation (Fig. 6e, Supplementary Data Fig. S6, Supplementary Table S1). These findings indicate that WIP1 promotes the tumor-initiating ability of NSCLC cells, while the tested WIP1 inhibitor suppresses this ability by abrogating and enhancing, respectively, p38 activity in vivo.

In addition to affecting the frequency of tumor initiation, both WIP1 and GSK also significantly impacted tumor growth. WIP1 overexpression increased tumor weight measured at the end of the study (Fig. 6b) and the growth rate of xenograft tumors over time (Fig. 6c, d), both of which were reversed by MKK3E (Fig. 6b–d). In contrast, GSK reduced tumor weight (Fig. 6f) and the tumor growth rate (Fig. 6g, h), and the reductions were abrogated by MKK6A (Fig. 6f–h).

We also performed H&E and immunohistochemical staining of xenograft tumor tissues formed by injection of 1 × 10^6^ H460 cells from each group to evaluate the CSC properties of these tumors. The results showed that overexpression of WIP1 increased the levels of the stemness-related protein SOX2 and the CSC marker ALDH1A1 in xenograft tumors, which could be reversed by MKK3E (Fig. 6i, Supplementary Data Fig. S7). In contrast, GSK treatment decreased the levels of SOX2 and ALDH1A1 in xenograft tumors, and these inhibitory effects could be abolished by MKK6A (Fig. 6j, Supplementary Data Fig. S8). Thus, WIP1 promotes and GSK suppresses tumorigenesis in NSCLC cells in a p38-dependent manner, likely through modulation of CSC properties in vivo.

## Discussion

Despite the tumor-suppressing functions of the p38 MAPK, mutations in p38 and altered p38 expression are rare in cancer. We previously reported that the activating phosphorylation of p38 is reduced in NSCLC tissues,^25^ suggesting that the activity of p38 is indeed downregulated during cancer development. In this study, we identified increased expression of WIP1, a p38 phosphatase, as at least one of the mechanisms underlying p38 inactivation in cancer. Furthermore, we demonstrated that by downregulating p38 activity, WIP1 promoted the expression of stemness-related transcription factors and CSC properties in NSCLC cells. These studies have thus identified a novel signaling pathway mediating the acquisition and maintenance of CSCs, which are the major source of tumor initiation, relapse, and drug resistance in cancer.

While the tumor-promoting functions of WIP1 and its overexpression in multiple human cancers have been reported previously, WIP1 oncogenic activity has been attributed to the suppression of p53.^26^ For example, WIP1 can directly dephosphorylate p53 at Ser15, whose phosphorylation is critical for the activation of p53. WIP1 can dephosphorylate MDM2, an E3 ubiquitin ligase of p53, leading to destabilization of the p53 protein. WIP1 also dephosphorylates and inactivates ATM, Chk1, and Chk2, which are upstream kinase activators of p53 that function in response to DNA damage. Our finding that WIP1 promotes CSC properties in NSCLC by inactivating p38 has revealed a novel mechanism underlying the oncogenic activity of WIP1. Importantly, this new oncogenic activity of WIP1 seems to be p53 independent, as WIP1 inhibited p38 phosphorylation and enhanced stemness-related protein expression and CSC properties, and the WIP1 inhibitor GSK2830371 reduced the expression of stemness-related transcription factors and the cancer stemness marker ALDH1 in H1299 cells with homozygous deletion of the TP53 gene (Figs. 2a, d–f and 5g).

WIP1 inhibitors, such as GSK2830371, are currently under development as anticancer drugs, based on their abilities to inhibit tumor growth and sensitize cancer cells to chemotherapy by reactivating p53.^27–29^ Therefore, WIP1 inhibitors will be effective only in cancer patients with wild-type p53. Our findings on the ability of WIP1 to promote CSC properties and that of a WIP1 inhibitor to suppress these properties in p53-deficient NSCLC cells have defined a new mechanism underlying the therapeutic efficacy of WIP1 inhibitors, and suggest that these inhibitors may have effects beyond p53, and be effective regardless of the p53 status in NSCLC patients with either wild-type or mutant p53. Moreover, since WIP1 overexpression also occurs in cancers other than NSCLC, WIP1 inhibitors may be useful in targeting CSCs in additional cancer types.

## Materials and methods

### Cell culture

A549, H1299, and H460 cells were obtained from ATCC, and cultured in RPMI-1640 medium containing 10% fetal bovine serum (FBS) (Biological Industries, USA) and 1% antibiotics (penicillin and streptomycin). All cells were maintained in a 5% CO_2_ incubator at 37 °C, and the medium was changed three times a week. When necessary, 10 μM GSK2830371 (Selleck, USA; S7573) was used to inhibit WIP1 during cell culture.

### Plasmid construction

The full-length CDS fragment of WIP1 was generated by polymerase chain reaction (PCR) and inserted into pLV-EF1-MCS-IRES-Bsd (Biosettia, USA). Single-strand oligos of shRNAs specific for WIP1 were synthesized, annealed into double-stranded oligos, and inserted into pLV-H1-EF1α-puro (Biosettia) following the guidance of the manufacturer’s protocol. DNA sequencing was then used to verify the plasmids. The human MKK3E and MKK6A plasmids were described previously.^39^ Primer sequences are provided in the Supplementary Materials (Table S2).

### Retrovirus- and lentivirus-based gene transduction

Retrovirus-mediated gene transduction was described previously. HEK293T cells were transfected with lentiviral vectors and packaging plasmids via Lipofectamine 2000 (Invitrogen, USA) to generate lentiviruses. Forty-eight hours after transfection, viral supernatants were collected, resuspended, and filtered through 0.45-μm filters (Millipore, USA). H1299, A549, and H460 cells were seeded in six-well plates and incubated with 2 ml of lentivirus-containing medium. The cells were then centrifuged at 1600 rpm at room temperature for 1 h. After centrifugation, the lentivirus-containing medium was discarded, and 3 ml of fresh complete RPMI-1640 medium was added to the wells. Forty-eight hours after transfection, 15 μg/ml blasticidin was added to the medium to select H1299-WIP1 and H460-WIP1 cells. A549-sh298, A549-sh1369, H460-sh298, and H460-sh1369 cells were selected with 0.8 μg/ml puromycin for 1 week to obtain stably transfected cell lines.

### RNA isolation, reverse transcription, and qRT-PCR analysis

Total RNA was isolated from samples using TRIzol reagent (Invitrogen), and cDNA was obtained using TransScript First-Strand cDNA Synthesis SuperMix (TransGen, China). TransStart Green Q-PCR SuperMix (TransGen) was used for quantitative real-time PCR (qRT-PCR) following the manufacturer’s protocol. Relative mRNA expression levels were normalized to GAPDH levels. The primer sets we used are shown in the Supplementary materials (Supplementary Table S2).

### Protein extraction and Western blotting

Total protein was collected using a protein extraction kit (CWBIO, China). A BCA protein assay kit (Pierce Biotechnology, USA) was used to determine the concentrations of proteins. Western blotting was performed as described previously.^40^ The antibodies used in this study included anti-WIP1 (Abcam, USA, ab31270), anti-p38 (Abcam, ab32142), anti-phospho-p38 MAPK (Cell Signaling Technology, USA, #4511), anti-MAPKAPK-2 (Cell Signaling Technology, #3042), anti-phospho-MAPKAPK-2 (Thr222) (Cell Signaling Technology, #3316), anti-phospho-MAPKAPK-2 (Thr334) (Cell Signaling Technology, #3041), anti-HSP27 (Cell Signaling Technology, #2402), anti-phospho-HSP27 (Ser82) (Cell Signaling Technology, #9709), anti-SOX2 (Proteintech, USA, 11064-1), anti-OCT4 (Proteintech, 11263-1), anti-NANOG (Proteintech, 14295-1), anti-ALDH1A1 (Santa Cruz, USA, sc-374149), and anti-β-actin (Santa Cruz, sc-47778). Antibodies against p38 isoforms (p38α, β, γ, and δ) have been described previously.^41^

### Sphere formation assay

H1299, A549, and H460 cells were prepared in single-cell suspensions with DMEM/F12 (Biological Industries) containing 20 ng/ml epidermal growth factor (EGF, Invitrogen), 20 μl/ml B27 (Invitrogen), 20 ng/ml basic fibroblast growth factor (bFGF, Invitrogen) and 1% antibiotics (HyClone, USA). Cells were seeded in 24-well ultralow-attachment plates (Corning, USA) at the concentration of 1 × 10^3^ cells/well. After incubating in a 5% CO_2_ incubator at 37 °C for 8–14 days, images were acquired using an inverted microscope fitted with a camera. Numbers and sizes of spheres were quantified in three separate, randomly chosen 100× fields.

### Side population assay

The percentage of the side population was determined in cell lines as described previously.^25^ Briefly, lung cancer cells cultured in six-well plates were incubated with 5 μg/ml Hoechst 33342 in PBS supplemented with 2% FBS at 37 °C with 5% CO_2_ for 1 h. Forty minutes before the addition of Hoechst 33342, 10 mM FTC (for A549) or 5 mM resperin (for H460 and H1299) was added to the control group to block the activity of Hoechst 33342. After staining, the cells were digested with Trypsin-EDTA and collected into 1.5-ml tubes. The cells were centrifuged at 1200 rpm at 4 °C for 8 min, and resuspended in 1 ml of cold PBS (containing 2% FBS). Finally, propidium iodide (PI) was added to the cell suspension at 1 μg/ml before flow cytometry analysis. The Hoechst 33342 dye was excited at 350 nm and measured at both 675 nm (Hoechst Red) and 450 nm (Hoechst Blue). The side population was gated for each group based on the proportion of cells that disappeared in the blocker control for the group.

### Hematoxylin and eosin (H&E) and immunohistochemical staining

Serial sections of a human lung tumor tissue array containing 116 NSCLC tumor tissue samples (60 squamous cell carcinoma and 56 adenocarcinoma specimens) and 16 normal/normal adjacent lung tissue samples (N/NAT) (4N and 12 NAT specimens) were purchased from Alenabio (LC121c and LC241h). H&E and immunohistochemical staining were performed according to previously described protocols,^42^ using a 1:1000 dilution of an anti-WIP1 antibody (Abcam, ab31270), 1:100 dilution of an anti-phospho-p38 MAPK antibody (Cell Signaling Technology, #4511), 1:100 dilution of an anti-SOX2 antibody (Proteintech, 11064-1), and 1:100 dilution of an anti-ALDH1A1 antibody (Santa Cruz, sc-374149). The images were acquired with an optical microscope fitted with a camera (Olympus, Japan). To evaluate the expression of WIP1, p-p38, SOX2, and ALDH1 in normal or tumor tissue samples, staining scores were evaluated by multiplying the positive staining area (scored as 1: 0–25%; 2: 26–50%; 3: 51–75%; 4: 76–100%) with the staining intensity (scored as 1: weak; 2: moderate; 3: strong; 4: very strong).

### Generation and analysis of xenograft tumors

All animal studies were conducted under approved protocols. Four- to six-week-old male BALB/c nude mice were purchased from Beijing HFK Bioscience Company. For the assessment of tumor-initiating ability in vivo, varying numbers (1 × 10^6^, 5 × 10^5^, 1 × 10^5^, and 6 × 10^4^) of H460-BP-pLV, H460-BP-WIP1, H460-MKK3E-pLV, H460-MKK3E-WIP1, H460-BH, or H460-MKK6A cells at an early passage were suspended in 100 μl of PBS and subcutaneously (s.c.) injected into the flanks of BALB/c nude mice. To determine the effects of GSK2830371 on tumor initiation and growth, mice injected with H460-BH or H460-MKK6A cells were randomly divided into two groups, one treated with a DMSO control and the other treated with 25 mg/kg GSK2830371 by intraperitoneal (i.p.) injection once daily from day 1 to 30. The length and width of tumors were measured every 4 days starting on day 10, and tumor volume was calculated with the following formula: Tumor volume = length × width^2^/2. The mice were sacrificed on the 31st day after the injection of tumor cells, and the tumors were harvested, weighed, and photographed. Subsequently, the tumors were dehydrated, embedded in paraffin, and sliced into 4-μm-thick tissue sections for H&E and immunohistochemical staining.

### Analysis of the TCGA database

The TCGA data for the two major subtypes of NSCLC, LUSC, and LUAD, were obtained from the cbioportal (www.cbioportal.org). The patients in each dataset were divided into WIP1-high (the top 25%, ≥75%) and WIP1-low (the bottom 25%, ≤25%) subgroups. The Mann–Whitney test was applied to assess the correlations between the expression levels of WIP1 and ALDH1 isoforms (A1–A3 and B1).

### Statistical analyses

Statistical analyses were performed using SPSS 20.0 (SPSS, USA), and data are generally expressed as the mean ± standard deviation. Spearman’s rank correlation analysis was performed to evaluate correlations in gene expression in the tissue array. Other data were compared by Student’s *t* test or the Mann–Whitney test. A *P* value less than 0.05 was considered statistically significant.

## Supplementary information

SUPPLEMENTAL MATERIAL
