# Supplementary material for: WIP1 promotes cancer stem cell properties by inhibiting p38 MAPK in NSCLC
Source: Signal Transduct Target Ther. 2020 Apr 15;5:36. doi: 10.1038/s41392-020-0126-x (PMC7156655; doi:10.1038/s41392-020-0126-x)
Supplement: Supplementary file 1 — SUPPLEMENTAL MATERIAL [file 41392_2020_126_MOESM1_ESM.docx]

Supplementary Materials for

**WIP1 Promotes Cancer Stem Cell Properties by Inhibiting p38 MAPK in NSCLC**

Kaiyuan Deng, Liang Liu, Xiaoming Tan, Zhen Zhang, Jianjun Li, Yang Ou, Xin Wang, Shuang Yang, Rong Xiang*, Peiqing Sun*

Correspondence to: rxiang@nankai.edu.cn；psun@wakehealth.edu

**This PDF file includes:**

Figures. S1 to S8

Tables S1 to S2


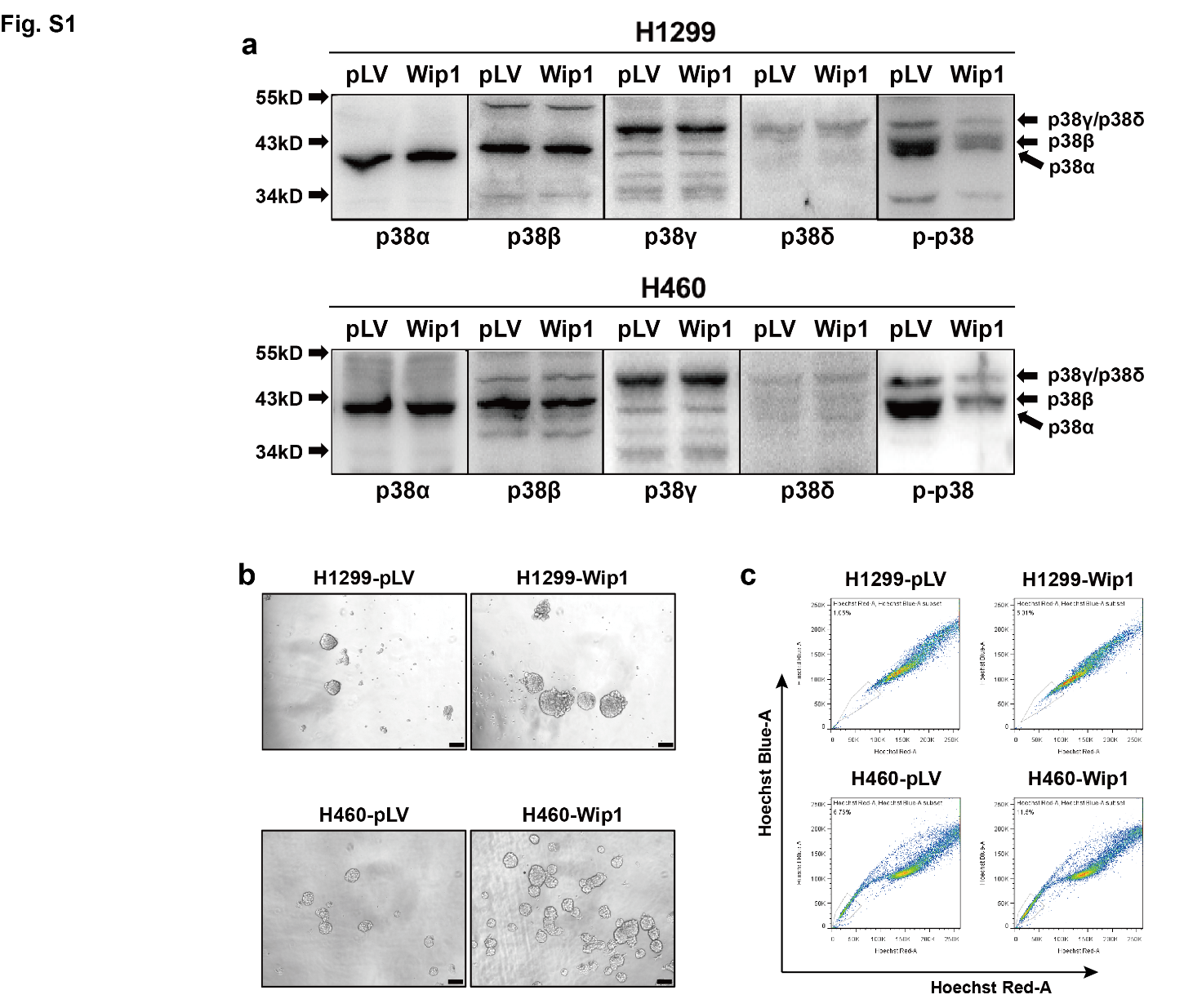


Figure. S1.

Ectopic expression of WIP1 increases the CSC properties in H1299 and H460 cells.


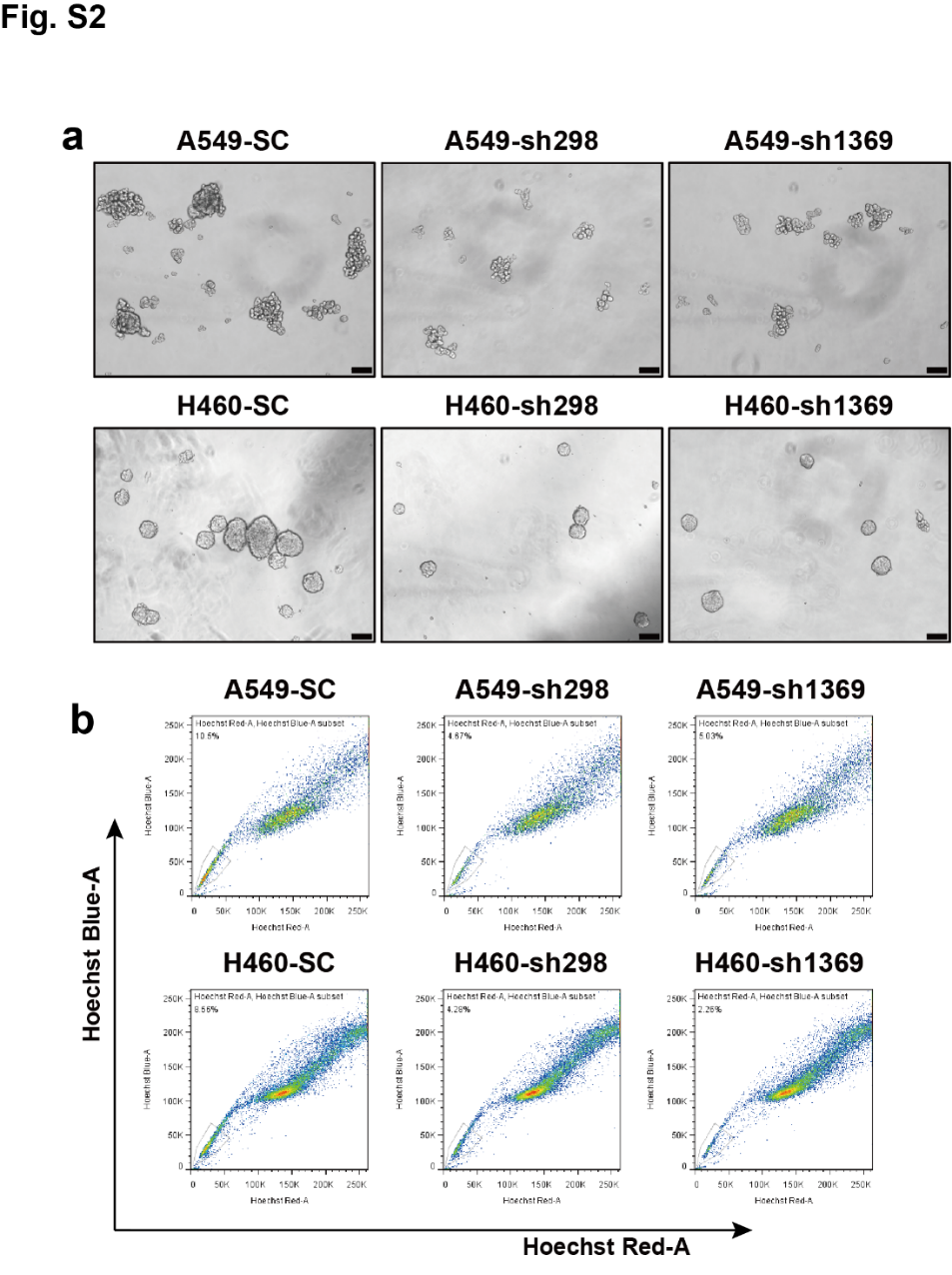


Figure. S2.

shRNA-mediated knockdown of WIP1 decreases the CSC properties in A549 and H460 cells.


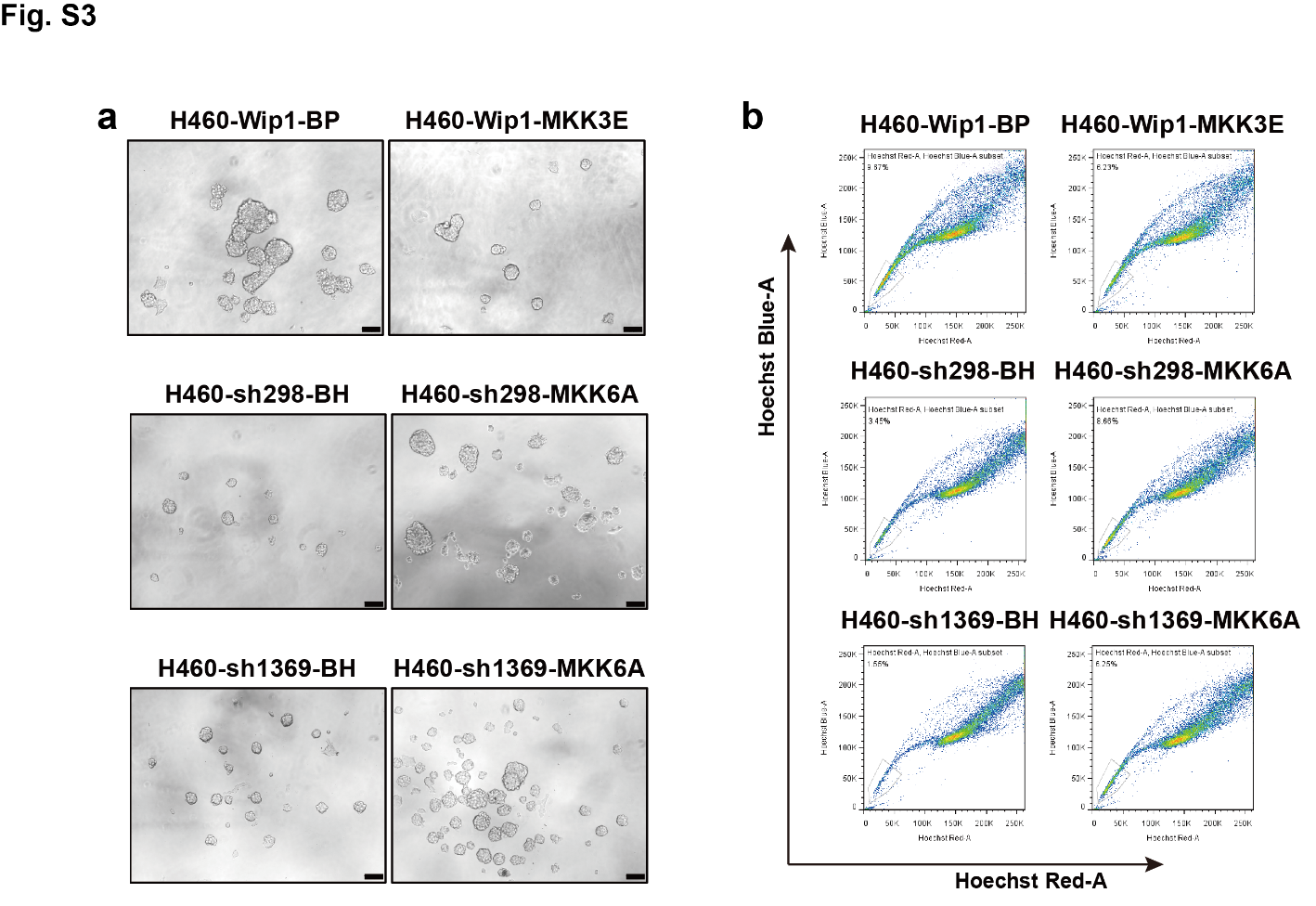


Figure. S3.

WIP1 enhances the CSC properties by inhibiting p38 activation in H460 cells.


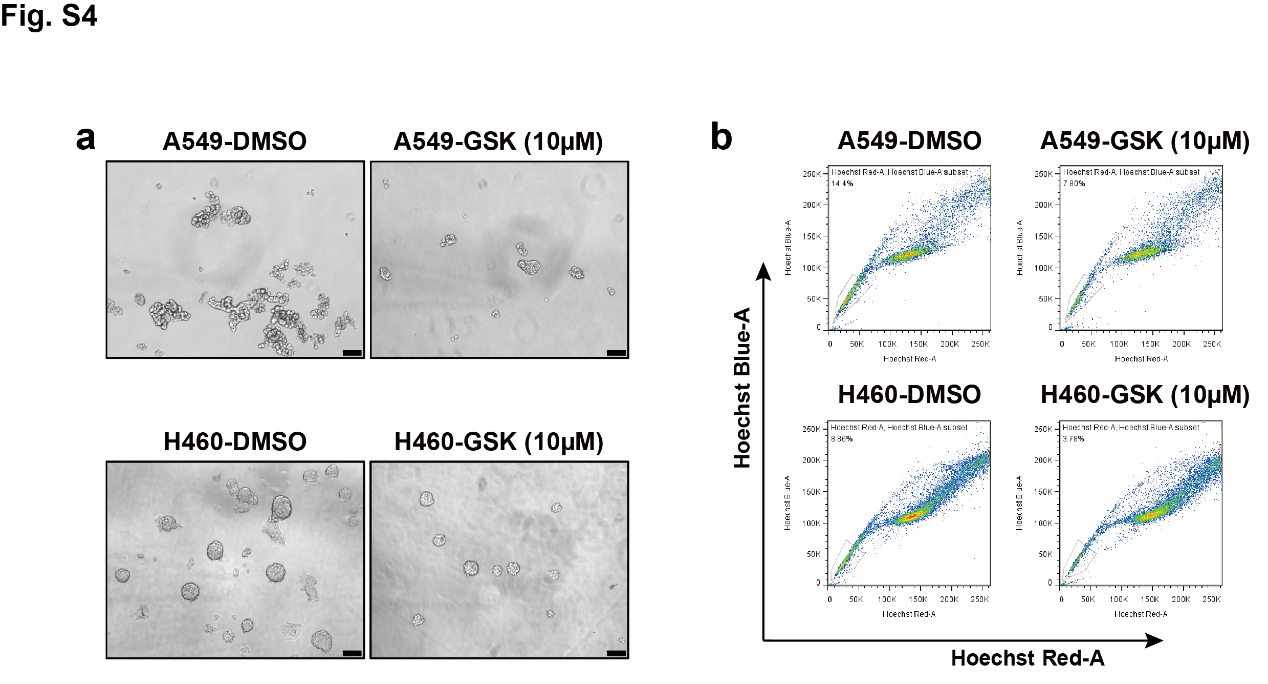


Figure. S4.

WIP1 inhibitor GSK2830371 inhibits the CSC properties in A549 and H460 cells.


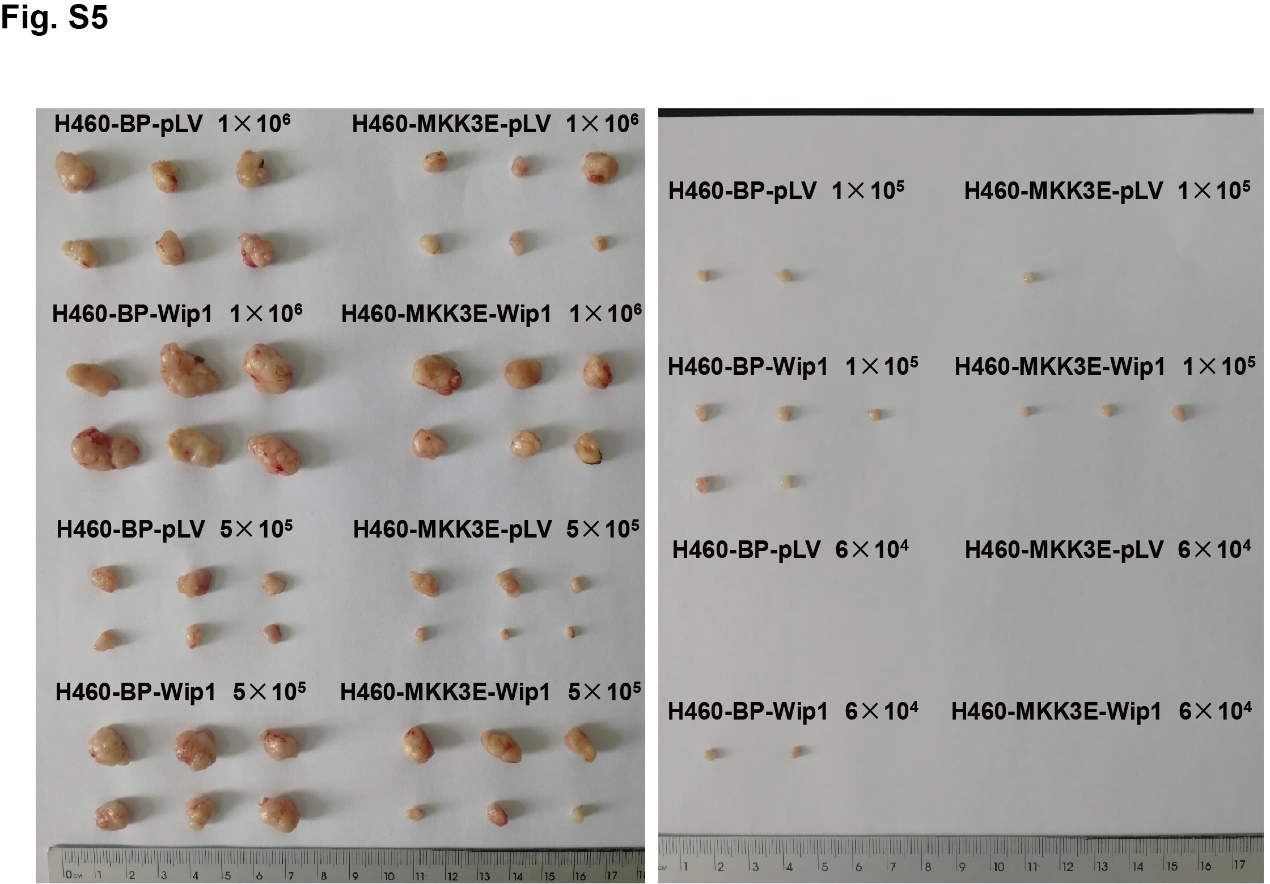


Figure. S5.

WIP1 promotes the tumor initiating ability of H460 cells and the growth rate of xenograft tumors formed by H460 cells in a p38-dependent manner.


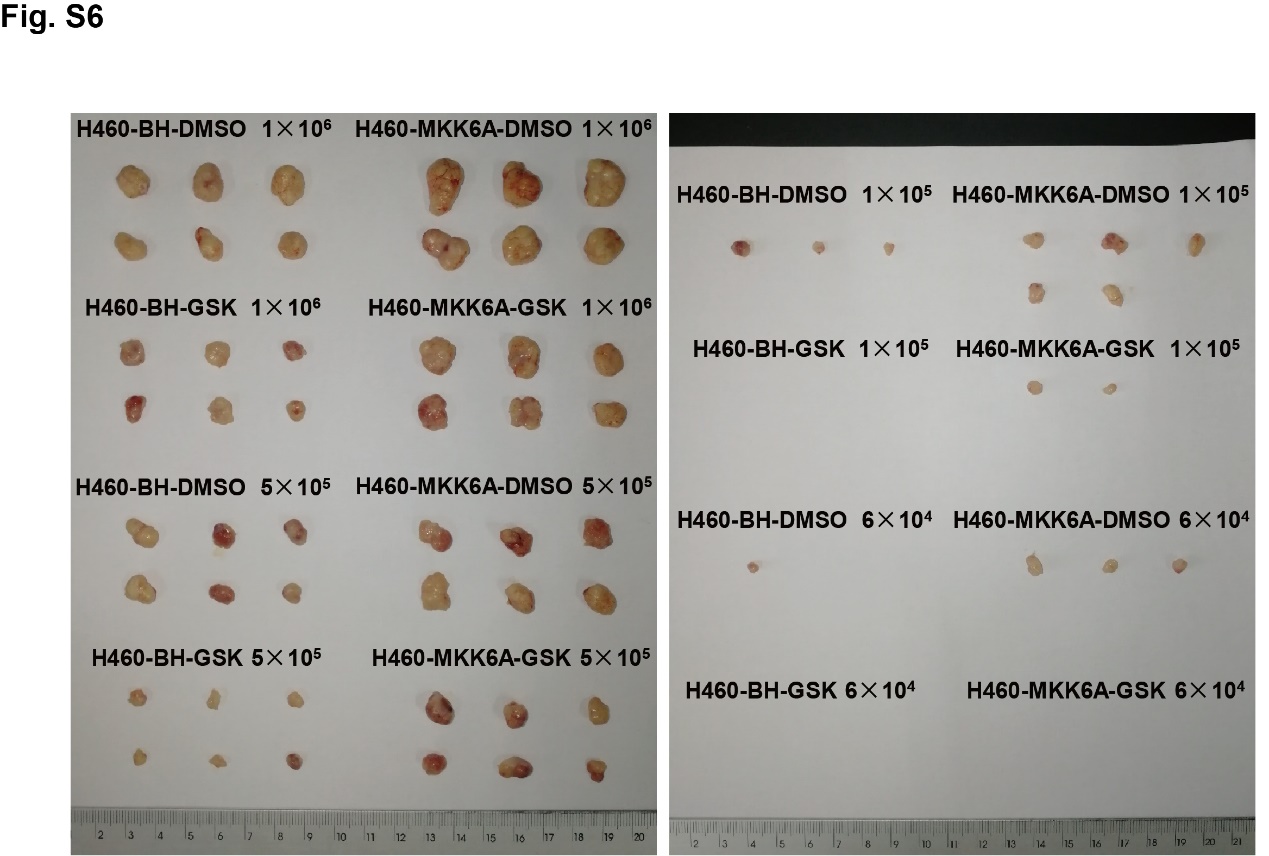


Figure. S6.

WIP1 inhibitor GSK2830371 suppresses the tumor initiating ability of H460 cells and the growth rate of xenograft tumors formed by H460 cells in a p38-dependent manner.


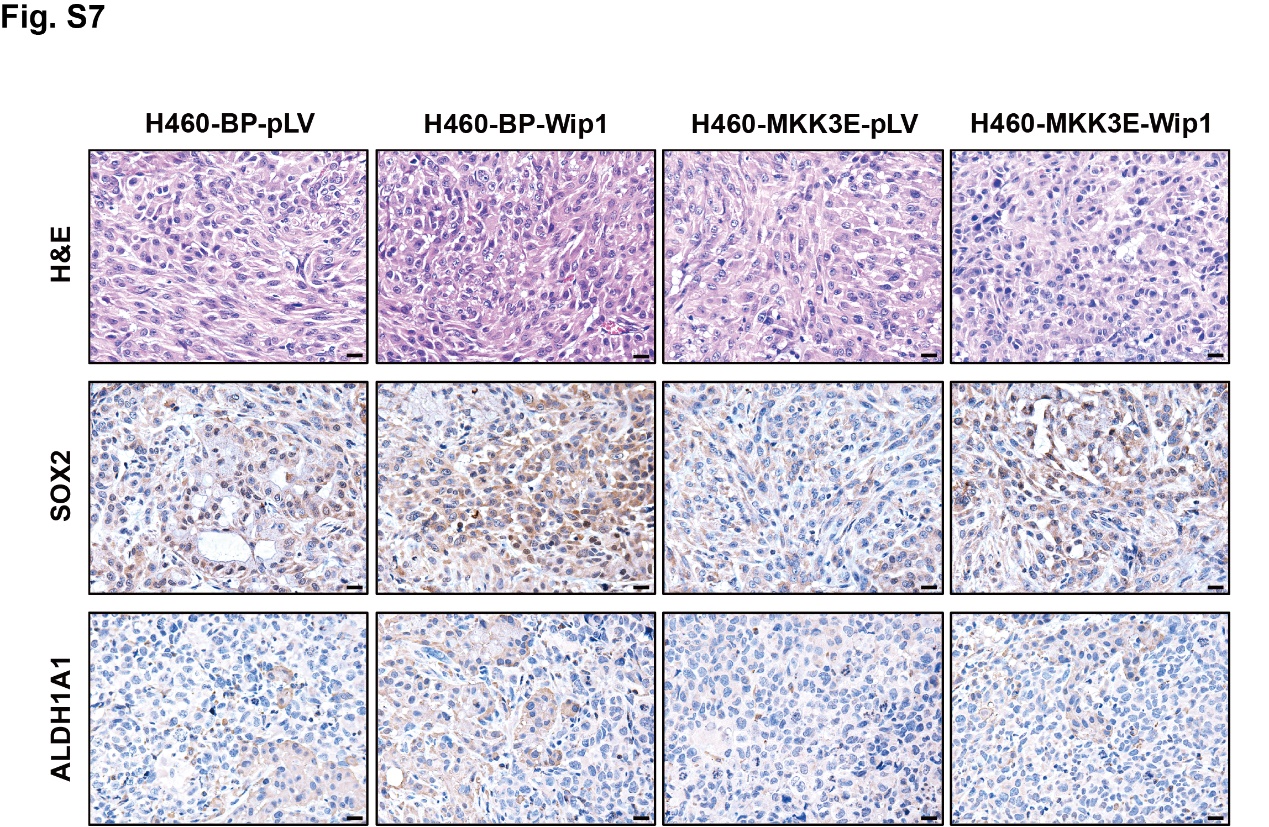


Figure. S7.

WIP1 increases the levels of the stemness protein and the CSC marker in xenograft tumors formed by H460 cells in a p38-dependent manner.


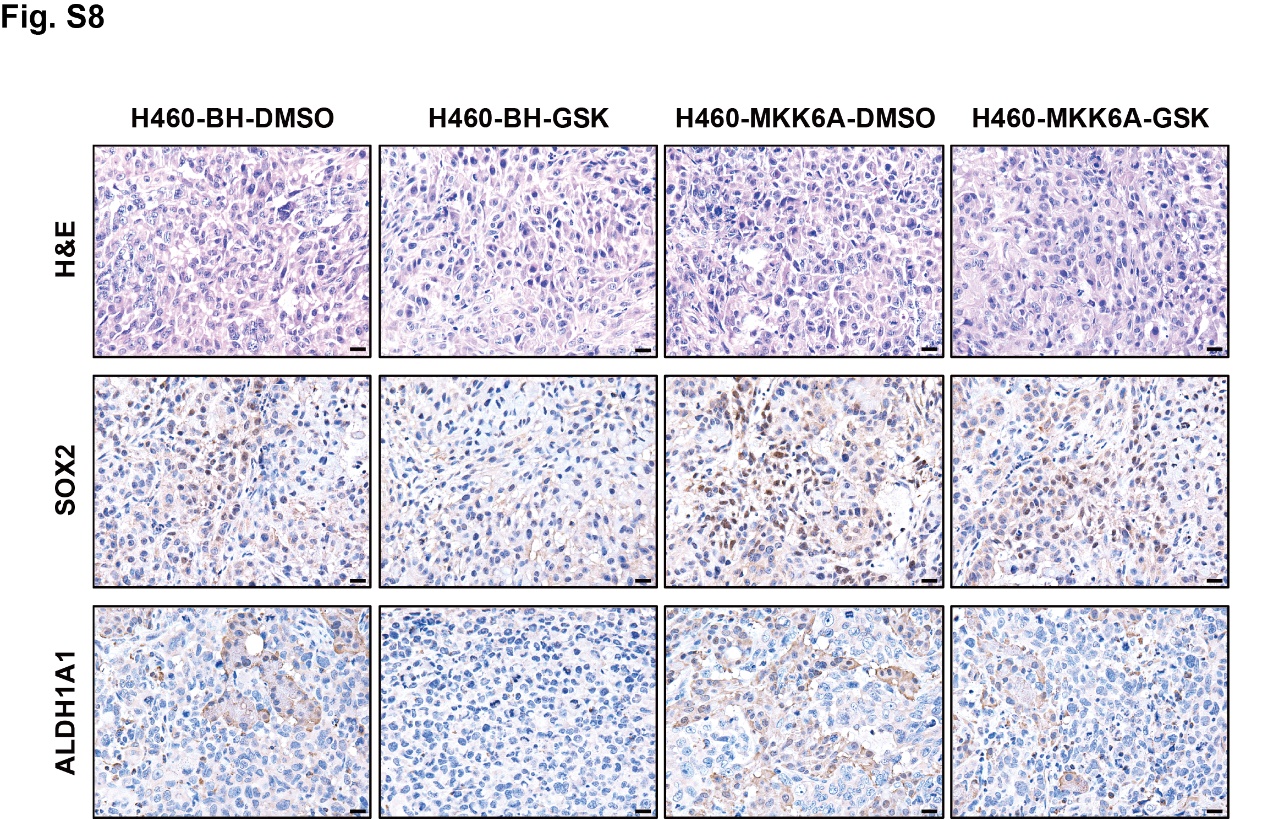


Figure. S8.

A WIP1 inhibitor GSK2830371 decreases the levels of the stemness protein and the CSC marker in xenograft tumors formed by H460 cells in a p38-dependent manner.

**Table S1. Tumor initiating capacity of xenograft tumors**

| **Group** | **Tumor Formation** | | | | **TIC Frequency^-1^** |
| --- | --- | --- | --- | --- | --- |
|  | 1 x 10^6^ | 5 x 10^5^ | 1 x 10^5^ | 6 x 10^4^ |  |
| **H460-BP** | 6/6 | 6/6 | 2/6 | 0/6 | 220478 |
| **H460-BP-Wip1** | 6/6 | 6/6 | 5/6 | 2/6 | 83796 |
| **H460-MKK3E** | 6/6 | 6/6 | 1/6 | 0/6 | 260312 |
| **H460-MKK3E-Wip1** | 6/6 | 6/6 | 3/6 | 0/6 | 184854 |
| **Group** | **Tumor Formation** | | | | **TIC Frequency^-1^** |
|  | 1 x 10^6^ | 5 x 10^5^ | 1 x 10^5^ | 6 x 10^4^ |  |
| **H460-BH** | 6/6 | 6/6 | 3/6 | 1/6 | 156211 |
| **H460-BH-GSK** | 6/6 | 6/6 | 0/6 | 0/6 | 305337 |
| **H460-MKK6A** | 6/6 | 6/6 | 5/6 | 3/6 | 68255 |
| **H460-MKK6A-GSK** | 6/6 | 6/6 | 2/6 | 0/6 | 220478 |

**Table S2. Primer information**

| Primer sequences | | |
| --- | --- | --- |
| Wip1-CDS | F: CGACGCGTGCCACCATGGCGGGGCTGTACTCG | R: CTAGCTAGCTCAGCAAACACAAACAGTTTTCC |
| Wip1-qPCR | F: CTGTACTCGCTGGGAGTGAG | R: GTTCGGGCTCCACAACGATT |
| GAPDH-qPCR | F: ACGGATTTGGTCGTATTGGGC | R: TTGACGGTGCCATGGAATTTG |
| Wip1-sh298 | AAAAGGACGTTACTCAAATCGTTTTGGATCCAAAACGATTTGAGTAACGTCC | |
| Wip1-sh1369 | AAAAGGACAATCAGGGAAACTTTATTGGATCCAATAAAGTTTCCCTGATTGTCC | |
